# Base edited “universal” donor CAR T-cell strategies for acute myeloid leukaemia

**DOI:** 10.1038/s41375-025-02720-5

**Published:** 2025-10-01

**Authors:** Renuka Kadirkamanathan, Christos Georgiadis, Arnold Kloos, Akshay Joshi, Annie Etuk, Roland Preece, Oliver Gough, Axel Schambach, Martin Sauer, Michael Heuser, Waseem Qasim

**Affiliations:** 1 UCL Great Ormond Street Institute of Child Health, London, UK; 2 Department of Hematology, Hemostasis, Oncology, and Stem Cell Transplantation, Hannover Medical School, Hannover, Germany; 3 Institute of Experimental Hematology, Hannover Medical School, Hannover, Germany; 4 Division of Hematology/Oncology, Boston Children’s Hospital, Harvard Medical School, Boston, MA USA; 5 Department of Pediatric Hematology and Oncology and Blood Stem Cell Transplantation, Hannover Medical School, Hannover, Germany; 6 Department of Internal Medicine IV, University Hospital Halle (Saale), Martin-Luther-University Halle-Wittenberg, Halle, Germany

**Keywords:** Immunotherapy, Cancer immunotherapy

## Abstract

Acute myeloid leukaemia (AML) is often aggressive and life-threatening with limited curative options. Immunotherapies including chimeric antigen receptor (CAR) T-cell approaches are under investigation, but high levels of disease heterogeneity remain a major hurdle to achieving durable responses. Targeting of multiple antigens may ensure complete immunological coverage of leukaemic blast populations, but such antigens are often also present on healthy haematopoietic populations. To address likely aplasia, strategies can be designed to bridge CAR T-cell therapies to allogeneic stem-cell transplantation (allo-SCT), as demonstrated in recent anti-CD7 CAR T-cell studies. Here we report that monotherapy using base edited “universal” donor CAR T cells against CD33, CLL-1, or CD7 delivered inhibition of AML in immunodeficient mice when antigen expression was homogenous, but combined use of BE-CAR33 and BE-CARCLL-1 T cells was required to address heterogenous CLL-1^-/+^CD33^-/+^ disease. We also demonstrate that removal of shared CD7 antigens enabled compatibility of BE-CAR33 and BE-CARCLL-1 with BE-CAR7 T cells, including in a patient-derived xenograft (PDX) model of AML. Therapeutic strategies envisage ‘pick and mix’ applications of base edited “universal” CAR T cells in combination determined by patient-specific antigen profiles. Such approaches also offer the possibility of deep, cell-based, de-bulking and conditioning ahead of allo-SCT and subsequent donor-derived reconstitution.

## Introduction

Acute myeloid leukaemia (AML) is often an aggressive, life-threatening disease with limited curative treatment options [1]. Newly diagnosed patients typically receive multiple rounds of chemotherapy and those with “high-risk” disease or elevated minimal residual disease (MRD) may undergo allogeneic haematopoietic stem cell transplant (allo-SCT) with the aim of harnessing graft-versus-leukaemia (GvL) effects [2]. Approaches using chimeric antigen receptor (CAR) T cells are also under investigation, but their development and application is more challenging than for B-cell acute lymphoblastic leukaemia (B-ALL), especially in the context of disease heterogeneity and reduced immune cell fitness. Targetable antigens include CD33, CLL-1, CD123, CD7, CD38 and FLT-3, but expression of these on healthy tissues as well as disease populations suggests immunotherapy must be time-limited to allow for eventual autologous recovery or donor derived reconstitution following haematopoietic SCT [3–12]. As an alternative to autologous CAR T cells, effectors can be generated from donors through an “off-the-shelf” approach, and use without HLA matching may be feasible following appropriate genome editing steps. We previously reported the use of cytidine base editing to develop “universal” donor CAR T cells (BE-CAR7) against CD7, a molecule expressed on T-cell acute lymphoblastic leukaemia (T-ALL) and present on a large proportion of healthy lymphocytes. Multiplexed knockouts prevented expression of αβ T-cell receptor (TCRαβ) to avoid graft-versus-host disease (GvHD), disrupted CD7 to evade self-targeting through the anti-CD7 CAR, and removed CD52 to confer resistance to the lymphodepleting antibody Alemtuzumab [13]. Similar anti-CD7 CAR T-cell products are being investigated for their ability to mediate lymphodepletion and myeloablation ahead of allogeneic (allo-) SCT, and have been utilised to treat AML exhibiting CD7 antigen expression [14]. However, the majority of AML blasts have heterogenous antigen expression, necessitating the targeting of multiple antigens simultaneously [15]. Combinational approaches against B-cell malignancies have investigated various strategies ranging from the expression of different CARs through the transduction of T cells using multiple vectors, as well as bi-cistronic constructs or tandem configurations [16, 17]. Our “off-the-shelf” approach envisages the co-infusion of cells from pre-generated CAR T-cell banks with different target specificities. We therefore manufactured universal BE-CAR formulations against CD33 (siglec-3) or C-type lectin-like molecule-1 (CLL-1/CLEC12A), both of which are commonly over-expressed in childhood and adult AML [3, 4]. Multiplexed base-editing of *TRBC*, *CD7* and *CD52* generated universal BE-CAR33 and BE-CARCLL-1 CAR T cells compatible with our existing BE-CAR7 products. Each BE-CAR product was investigated alone, or in combination, against homogenous and heterogenous cell lines, as well as primary AML, as a cell-based universal CAR T-cell immunotherapy approach against AML.

## Methods

### Cell lines and primary cells

HEK293Ts, Kasumi-3, HL-60 and MOLM-14 cell lines were obtained from ATCC; U-937 were kindly gifted by Prof. Owen Williams GOS ICH, UCL, UK. Peripheral blood mononuclear cells (MNC) were obtained as steady state leukapheresis collections from registry donors (Antony Nolan) or blood samples from volunteer donors at University College London (UCL). Patient-derived xenograft (PDX) AML models were established at Hannover Medical School (MHH), Germany from a subject with t(3;3) translocation involving *EVI1*.

### Preparation of 3^rd^ generation BE-CAR lentiviral vectors

pCCL viral vector encoding BE-CAR7 was generated as described [13]. CAR33 and CARCLL-1 constructs incorporated codon-optimised scFv sequences from variable-heavy and variable-light chain regions of My96 anti-CD33 (DrugBank ID DB00056) and M26 anti-CLL-1 (US 2013/0295118) clones, respectively. All CARs included a CD8 derived transmembrane region and 4-1BB and CD3ζ activation domains under the control of a phosphoglycerate kinase (PGK) promoter. pTTB-CAR33 and pTTB-CARCLL-1 viral vectors also incorporated a sgRNA expression cassette against *TRBC* embedded in the 3’ long terminal repeat (LTR), similar to pTTB-CAR19 used previously [18]. Self-inactivating (SIN) lentiviral vector stocks were produced by transient transfection of HEK293T cells using 3^rd^ generation packaging plasmids (Plasmid Factory, Bielefeld, Germany) and pseudotyped with vesicular stomatitis virus G (VSV-G) envelope.

### Generation of BE-CAR T cell effectors

Mononuclear cells (MNC)s were activated with TransAct (Miltenyi Biotec, Bergisch Gladbach, Germany) and cultured in TexMACS (Miltenyi Biotec) media with 3% heat-inactivated human serum (Seralab, Sussex, UK) and 20 ng/mL human recombinant IL-2 (Miltenyi Biotec). BE-CAR7 and CAR19 T cells were generated as described previously [13, 19]. BE-CAR33 and BE-CARCLL-1 products were transduced 24 h after activation and electroporated a further 72 h later with sgRNAs against *CD7*, *CD52*, & *TRBC* (Synthego, Redwood City, CA, USA) and coBE3 (BioNTech, Mainz, Germany) using 4D-Nucleofector (Lonza, Basel, Switzerland, EW138 pulse code). Cells were maintained for 16 h at 30°C and then returned to 37°C in G-Rex chambers (Wilson Wolf, MN, USA) before magnetic TCRαβ depletion (Miltenyi Biotec, Germany) with overnight culture prior to cryopreservation.

### Flow-cytometry cytotoxicity assays

For compatibility studies, BE-CAR33 targets were labelled with CFSE and co-cultured with BE-CAR7 effectors at an E:T of 1:1 for 16 h. Cells were then stained with anti-CD2 antibody and DAPI and BE-CAR7 cytotoxicity was assessed through flow cytometry. Separately, BE-CAR7 cells were co-cultured with CD7^+^GFP^+^ Jurkat targets as a killing control. For cytotoxicity studies, PDX AML cells were co-cultured with CFSE labelled BE-CAR33, BE-CARCLL-1 or untransduced effectors at an E:T of 10:1 for 16 h. Live CFSE^-^CD45^dim^ PDX cells were then counted through flow cytometry using Precision Count Beads (Biolegend, San Diego, CA, USA).

### Radio-isotope cytoxicity assessments

Targets were loaded with ^51^Cr (PerkinElmer, Waltham, MA, USA) and co-cultured with untransduced cells or BE-CAR effectors at E:T ratios of 20:1 to 0.156:1 for 4 h before processing for microplate scintillation (Wallac 1450 MicroBeta TriLux, Perkin Elmer). Spontaneous and maximum ^51^Cr release was quantified after culture with cell media or lysis with 10X Triton X-100 (Sigma Aldrich, St. Lois, MO, USA), respectively. Specific lysis was calculated as [(experimental release – spontaneous release)/(maximum release – spontaneous release)] x 100%.

### In vivo BE-CAR activity against AML cell lines

Six-week-old NOD/SCID/γc^-/-^ (NSG) mice (Charles River strain 005557, Jackson Laboratory, Bar Harbor, ME, USA) were injected with 1×10^6^ Kasumi-3 GFP^+^LUC^+^ targets or 1×10^6^ HL-60 GFP^+^LUC^+^ targets (with a homogenous CLL-1^+^CD33^+^ or heterogenous CD33^-^CLL-1^+^/CD33^+^CLL-1^-^ phenotype). Tumour engraftment was measured on day 3 using bioluminescent imaging (BLI) (IVIS Lumina III, Revvity, Waltham, MA, USA). Mice received effectors on day 4 by tail vein injection and leukaemia progression was tracked until mice were euthanised. Bone marrow samples were harvested and processed for flow cytometry.

### In vivo BE-CAR activity in a PDX model of AML

Eight-to-twelve-week-old NOD/SCID/γc^-/-^ NSG mice were sub lethally irradiated at 2.5 Gy on day -21 and subsequently engrafted with 1×10^6^ primary AML cells by tail vein injection on day -20. Mice received effectors alone or both in combination on day 0 and disease progression was tracked through serial peripheral bleeds. Bone marrow samples were harvested and processed for flow cytometry after mice were euthanised.

### Statistical analysis

Significance was determined using one-way ANOVA with Tukey multiple comparison post-hoc test, unpaired T test or Mann-Whitney U test using GraphPad (La Jolla, CA, USA) Prism software V9.0. Error bars represent standard error of the mean.

## Results

### Generation of universal BE-CAR T cells

**Fig. 1:**
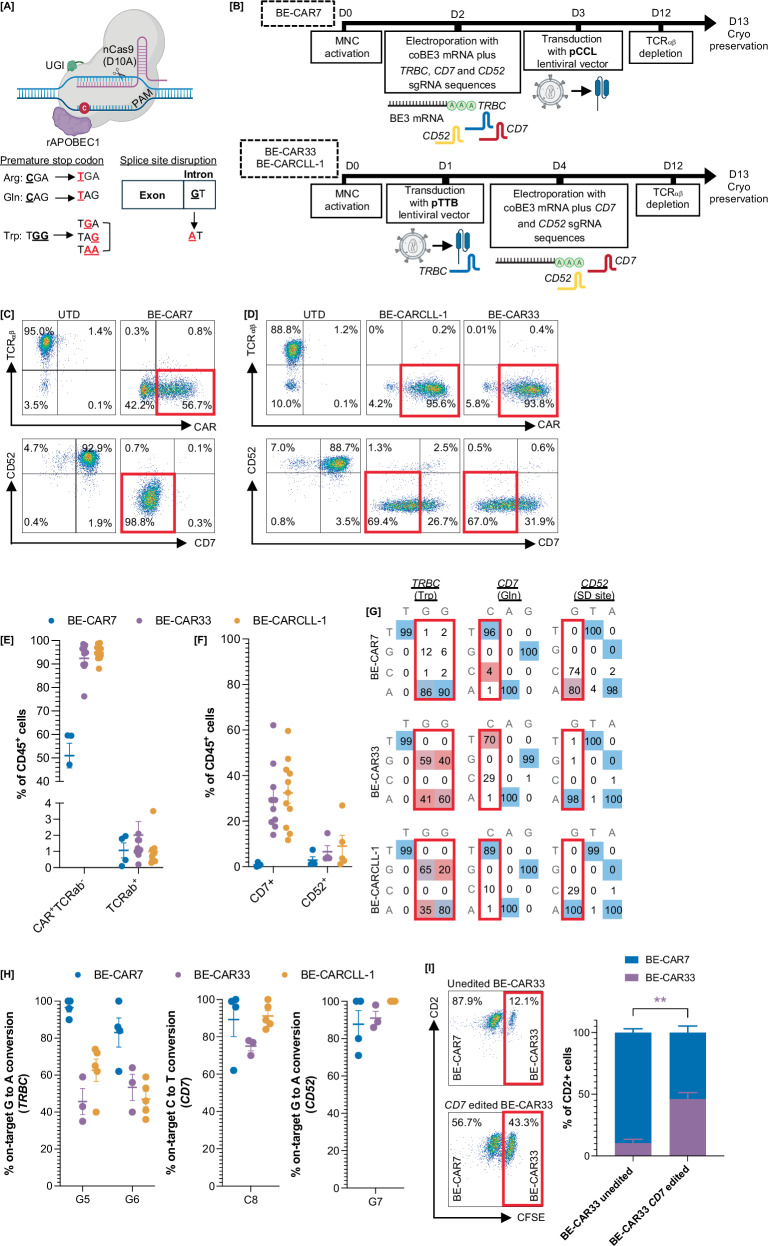
BE-CAR7, BE-CAR33 and BE-CARCLL-1 T cells to target AML. **A** Strategies employed to cause protein knockout using a cytidine base editor, through the C-to-T mediated conversion of arginine (arg), glutamine (gln) or tryptophan (trp) amino acid residues to premature stop codons (TGG, TGA, TAG), or the disruption of consensus sequences required for proper mRNA splicing. Created with BioRender.com. **B** Schematics of BE-CAR manufacture. For BE-CAR7 T cells, MNCs were electroporated with codon optimised mRNA encoding the cytidine base editor BE3 and sgRNAs against *TRBC* (introducing a premature stop codon), *CD7* (introducing a premature stop codon) and *CD52* (disrupting a splice donor site). Cells were then transduced with a pCCL-CAR7 and expanded prior to TCRɑβ magnetic column depletion. For BE-CAR33 and BE-CARCLL-1 T cells, MNCs were first transduced with pTTB lentiviral vector encoding anti-CD33 or anti-CLL-1 CAR and *a* sgRNA expression cassette against *TRBC*. coBE3 mRNA and sgRNAs were delivered by electroporation and residual TCRɑβ^+^ cells were removed through magnetic bead depletion. Created with BioRender.com. **C** Example of end-of-manufacture CD45^+^ BE-CAR7, **D** BE-CAR33 and BE-CARCLL-1 T cells with CAR^+^TCRαβ^-^ and CD7^-^CD52^-^ populations in red. **E** Summary of multiple donor BE-CAR7 (*n* = 4), BE-CAR33 (*n* = 10) and BE-CARCLL-1 (*n* = 11) T cells with CAR^+^TCRαβ^-^ phenotype and residual TCRαβ, **F** CD7 and CD52 expression. **G** Example of EditR molecular analysis of on-target base conversion at *TRBC*, *CD7* and *CD52* editing sites with **H** percentage of on-target conversions from multiple healthy donor BE-CAR7 (*n* = 4), BE-CAR33 (*n* = 3) and BE-CARCLL-1 (*n* = 5) T cells. **I** Co-culture of BE-CAR7 T cells and BE-CAR33 T cells (with and without CD7 expression) indicating evasion of BE-CAR7 fractricidal effects after CD7 base editing. ** *p* < 0.01 (unpaired *t* test).

BE-CAR7 T cells were generated as described previously using codon optimised (co)BE3 and sgRNAs targeting *CD7*, *TRBC* and *CD52* ahead of lentiviral pCCL-CAR7 transduction to prevent CAR7-mediated fratricide against CD7-expressing T cells (Figs. 1A, B and S1A), and resulted in 51.0 ± 5.3% BE-CAR7^+^TCRαβ^-^ cells (*n* = 4) (Fig. 1C, E) [13]. As CD33 and CLL-1 expression remains low, even on activated T cells, universal BE-CAR33 and BE-CARCLL-1 T cells provided high yields if generated by transducing T cells ahead of editing (Figs. 1B, S1A–D). Editing operations were transient, occurring only after coBE3 mRNA delivery by electroporation and in combination with sgRNAs targeting *CD7, CD52, or TRBC* (Fig. S1E). CAR expression at the end of production, following magnetic bead depletion of residual TCRαβ^+^ cells, was 92.4 ± 2.1% for CAR33^+^TCRαβ^-^ cells (*n* = 10) and 94.9 ± 1.0% for CARCLL-1^+^TCRαβ^-^ cells (*n* = 11) (Fig. 1D, E). Residual TCRαβ^+^ expression was 1.1 ± 0.5% (*n* = 4), 2.0 ± 0.8% (*n* = 10) and 1.0 ± 0.3% (*n* = 11) for BE-CAR7, BE-CAR33 and BE-CARCLL-1 products respectively (Fig. 1C–E). BE-CAR7 T cells underwent self-enrichment which resulted in <1% CD7^+^ cells (*n* = 4), whilst CD7 expression was documented on 29.8 ± 4.6% (*n* = 10) and 32.4 ± 4.5% (*n* = 11) end-of-manufacture BE-CAR33 and BE-CARCLL-1 T cells, respectively, and CD52 expression was present on 2.9 ± 1.5% (*n* = 4), 6.6 ± 2.7% (*n* = 4) and 9.0 ± 4.8% (*n* = 5) BE-CAR7, BE-CAR33 (*n* = 10) and BE-CARCLL-1 (*n* = 11) cells, respectively (Fig. 1C, D, F). Phenotyping of end-of-manufacture BE-CAR products captured CD45RA and CD62L expression to broadly define naïve (T_N_), central memory (T_CM_), effector memory (T_EM_) and terminally differentiated (T_EMRA_) T-cell subsets (Fig. S2A), whilst exhaustion marker profiles were provided through assessment of PD-1, BTLA, TIM-3 and/or LAG-3 expression (Fig. S2B).

Molecular mapping performed by Sanger sequencing revealed on-target G_5_ > A conversion for *TRBC* as 96.5 ± 2.4% for BE-CAR7 (*n* = 4), 45.7 ± 7.1% for BE-CAR33 (*n* = 3) and 62.6 ± 6.1% for BE-CARCLL-1 (*n* = 5) (Fig. 1G, H). At the *CD7* site, on-target C_8_ > T conversions were quantified as 89.3 ± 9.1% for BE-CAR7 (*n* = 4), 75.0 ± 2.5% for BE-CAR33(*n* = 3) and 91.2 ± 3.0% for BE-CARCLL-1 (*n* = 5), whilst on-target G_7_ > A conversions at the *CD52* site were 87.8 ± 7.3% for BE-CAR7 (*n* = 4), 91.0 ± 3.6% for BE-CAR33 (*n* = 3) and 100 ± 0% for BE-CARCLL-1 T cells (*n* = 3) (Fig. 1G, H). Digital droplet (dd)PCR was used to confirm vector copy number (VCN) (Fig. S2C).

Protective effects of *CD7* disruption on BE-CAR33 was confirmed in a 16-h co-culture assay where BE-CAR33 cells unedited for CD7 were targeted by BE-CAR7 cells, whereas CD7 edited BE-CAR33 cells largely evaded cytotoxicity (*p* < 0.01) (Figs. 1I, S2D).

### BE-CAR T cells against AML with homogenous antigen profiles

**Fig. 2:**
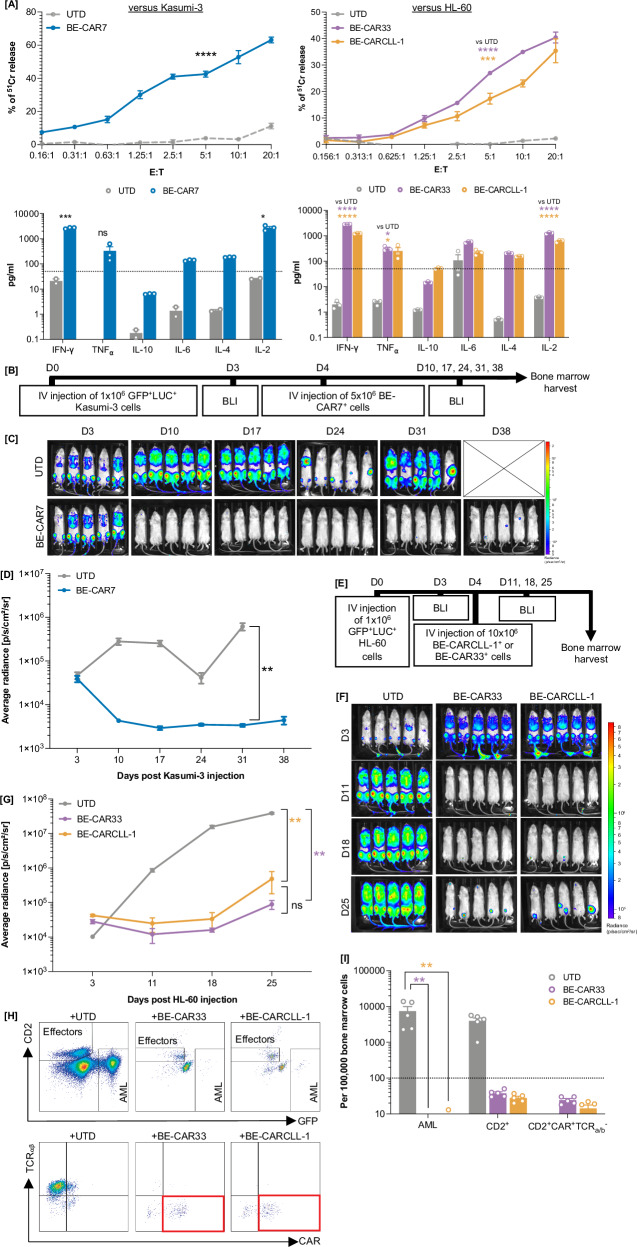
BE-CAR T cells exhibited antigen specific effects against AML with homogenous antigen expression. **A** (Top) ^51^Cr release from Kasumi-3 (left) or HL-60 (right) targets after co-culture with untransduced T cells or BE-CAR7, BE-CAR33 or BE-CARCLL1 effectors across E:T ratios. (Bottom) Cytokines released from untransduced, BE-CAR7, BE-CARCLL-1 or BE-CAR33 T cells (above 50 pg/mL as indicted by the dotted line) when co-cultured with Kasumi-3 (left) or HL-60 (right) targets at an E:T of 1:1. * *p* < 0.05, *** *p* < 0.001, **** *p* < 0.0001. (unpaired T test for BE-CAR7 comparison, one-way ANOVA with Tukey multiple comparison post-hoc for BE-CAR33 and BE-CARCLL-1 comparisons). **B** BE-CAR7 anti-leukaemic effects were assessed in vivo aginst a humanised murine model of AML using GFP^+^LUC^+^ Kasumi-3 cells. **C** Bioluminescent images and average radiance values (p/s/cm^2^/sr) **D** of mice that received 1×10^6^ Kasumi-3 targets on day 0 followed by 10×10^6^ untransduced T cells (*n* = 5) or 5 × 10^6^ BE-CAR7^+^ effectors (from 10 × 10^6^ total MNCs) (*n* = 5) on day 4. ** *p* < 0.01 (one-way ANOVA of AUC with Tukey multiple comparison post-hoc between D3 and D31). **E** BE-CAR33 and BE-CARCLL-1 anti-leukaemic effects were assessed in vivo against a humanised murine model of AML using GFP^+^LUC^+^ HL-60 targets. **F** Bioluminescent images and average radiance values (p/s/cm^2^/sr) **G** of mice that received 1 × 10^6^ HL-60 targets on day 0 followed by 10 × 10^6^ untransduced cells (*n* = 5), 10 × 10^6^ BE-CAR33^+^ (*n* = 5) or 10 × 10^6^ BE-CARCLL-1^+^ (*n* = 5) effectors (from 10×10^6^ total MNCs) on day 4. ** *p* < 0.01 (one-way ANOVA of AUC with Tukey multiple comparison post-hoc between D3 and D25). **H** CD45^+^GFP^+^ AML and CD45^+^CD2^+^/CD2^+^CAR^+^TCRαβ- effectors (highlighted in red) detected in bone marrow of mice from each group. **I** Normalised numbers of CD45^+^GFP^+^ AML cells and CD2^+^/CD2^+^CAR^+^TCRαβ- cells detected in bone marrow of mice (*n* = 5) from each treatment group. Limit of quantification is shown at 100 events per 100,000 bone marrow cells. ** *p* < 0.01 (one-way ANOVA with Tukey multiple comparison post-hoc).

Functional studies were performed to assess the in vitro cytotoxicity of BE-CAR7 T cells against Kasumi-3 cells, whilst BE-CAR33 and BE-CARCLL-1 T cells were tested against HL-60, MOLM-14 and U-937 cells and primary human AML samples expressing CD33 and CLL-1 (Fig. S3A, B). CD33 and CLL-1 antigen density was measured for each relevant cell line before ^51^Cr release cytotoxicity assays and cytokine release quantification after co-culture with effectors across a range of E:T ratios (Fig. S3C). Robust CAR-mediated target cell lysis was documented for each BE-CAR product, and cytokine release profiles captured (Figs. 2A, S3D, E). In addition to cell lines, BE-CAR33 and BE-CARCLL-1 targeting of CD33^high^CLL-1^high^ primary human AML was demonstrated in overnight co-culture experiments (Fig. S3F).

Next, the in vivo anti-leukaemia effects of each BE-CAR were assessed in a humanised xenograft murine model of AML. For BE-CAR7 T cells, 6-week-old NSG mice received 1 × 10^6^ GFP^+^LUC^+^ Kasumi-3 cells (*n* = 10) by tail-vein injection on day 0, and BLI was used to confirm disease engraftment on day 3. AML-bearing mice went onto receive 5 × 10^6^ BE-CAR7^+^ T cells (*n* = 5) or 10 × 10^6^ untransduced cells (*n* = 5) on day 4 with serial BLI performed on days 10, 17, 24, 31 and 38 to monitor disease progression (Fig. 2B, C). Potent leukaemia clearance was observed in mice treated with BE-CAR7 T cells compared to untreated mice (*p* < 0.01) (Fig. 2D). Separately, for BE-CAR33 and BE-CARCLL-1 T cells, 6-week-old NGS mice received 1 × 10^6^ GFP^+^LUC^+^ HL-60 cells (*n* = 15) by tail-vein injection on day 0, and BLI confirmed engraftment on day 3. AML-bearing mice went on to receive 10 × 10^6^ BE-CAR33^+^ (*n* = 5) or BE-CARCLL-1^+^ (*n* = 5) T cells, or untransduced cells (*n* = 5) on day 4 with serial BLI on days 11, 18 and 25 (Fig. 2E, F). Leukaemia progression was again inhibited in mice treated with either BE-CARs compared to untreated mice (*p* < 0.01), with scant GFP^+^LUC^+^ AML cells detected in bone marrow of treated mice compared to controls (*p* < 0.01) (Fig. 2H, I). BE-CAR33 and BE-CARCLL-1 cell persistence was also documented, with CD45^+^CD2^+^ effectors maintaining their CAR^+^TCRαβ^-^ phenotype (Fig. 2H, I).

### BE-CAR33 and BE-CARCLL-1 T cells against heterogenous AML

**Fig. 3:**
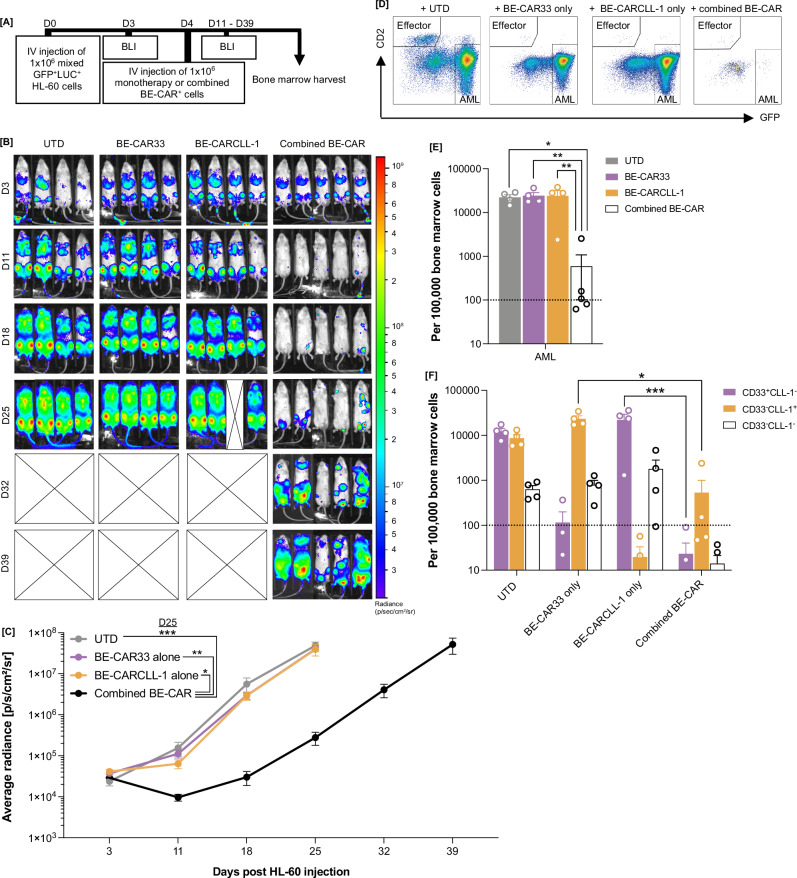
BE-CAR33 and BE-CARCLL-1 effectors combined effects against heterogenous AML in vivo. **A** Humanised xenograft model of heterogenous AML with GFP^+^LUC^+^ variants used to assess a combined dosing strategy with BE-CAR33 and BE-CARCLL-1 effectors. **B** Bioluminescent images and average radiance values (p/s/cm^2^/sr) **C** of mice that received 1 × 10^6^ CD33^-/+^CLL-1^+/-^ HL-60 targets on day 0 and 10 × 10^6^ untransduced T cells (*n* = 4), 4 × 10^6^ monotherapy BE-CAR33^+^ (*n* = 4) or 4 × 10^6^ BE-CARCLL-1 (*n* = 4) effectors, or 2 × 10^6^ BE-CAR33^+^ and 2 × 10^6^ BE-CARCLL-1^+^ combined effectors (*n* = 4) on day 4 (from a maximum of 10 × 10^6^ MNCs). * *p* < 0.05, ** *p* < 0.01, *** *p* < 0.001 (one-way ANOVA with Tukey multiple comparison post-hoc). **D** CD45^+^GFP^+^ AML targets and CD45^+^CD2^+^ effectors detected in bone marrow of mice from each group. **E** Normalised total CD45^+^GFP^+^ AML cell counts and **F** AML cell counts displaying CD33^+^CLL-1^-^, CD33^-^CLL-1^+^ or CD33^-^CLL-1^-^ phenotypes detected in bone marrow of mice (*n* = 4) from each treatment group. Limit of quantification is shown at 100 events per 100,000 bone marrow cells. * *p* < 0.05, ** *p* < 0.01 (3E: one-way ANOVA with Tukey multiple comparison post-hoc, 3F: Mann-Whitney U test, * *p* < 0.01, *** *p* < 0.001).

Combined targeting of CD33 and CLL-1 could offer a valuable strategy against heterogenous disease as both antigens are often upregulated on AML, although complete expression of either may not be complete across entire blast populations to enable monotherapy approaches. We therefore investigated use of BE-CAR33 in combination with BE-CARCLL-1 T cells in vivo against xenografted human CD33^+/-^ CLL-1^+/-^ AML. Mice were engrafted with equal numbers of variant HL-60 lines edited using CRISPR/Cas9 to express either CD33 (CD33^+^CLL-1^-^) or CLL-1 (CLL-1^+^CD33^-^) (Figs. 3A, B and S4A, B). BLI confirmed tumour engraftment on day 3 and AML-bearing mice went on to receive tail-vein injections of 10 × 10^6^ untransduced T cells (*n* = 4) or effector cells comprising 4 × 10^6^ BE-CAR33^+^ (*n* = 4) or 4 × 10^6^ BE-CARCLL-1^+^ T cells (*n* = 4) alone, or 2×10^6^ BE-CAR^+^ cells from each product combined (*n* = 4). Disease clearance was similar between 1-4×10^6^ BE-CAR33^+^ or BE-CARCLL-1^+^ effectors when used as monotherapy (Fig. S4C–H). Untreated mice and those receiving monotherapies exhibited significant disease progression by day 25 when compared with animals treated with combined BE-CAR effectors (Fig. 3C). Mice from monotherapy and untreated groups were euthanised on day 25 due to leukaemia progression, with high levels of bone marrow disease detectable by flow cytometry (Fig. 3D, E). Mice receiving BE-CAR33 or BE-CARCLL-1 monotherapies were only able to inhibit disease expressing their corresponding target antigen with resistant disease predominantly CD33^-^CLL-1^+^ or CD33^+^CLL-1^-^ respectively (*p* < 0.05, *p* < 0.001) (Figs. 3F, S3I). In contrast, mice receiving combined BE-CARs exhibited potent clearance of bone marrow disease, measured through BLI, compared to monotherapy groups (*p* < 0.01) and untreated controls (*p* < 0.05), which was coupled with minimal levels of dual target antigen loss (Fig. 3D–F). These findings together demonstrate combined anti-leukaemia effects of BE-CARCLL-1 and BE-CAR33 T cells can inhibit heterogenous AML more effectively than monotherapy strategies.

### Evasion of BE-CAR7 fractricidal activity by BE-CAR33 or BE-CARCLL1 effectors

**Fig. 4:**
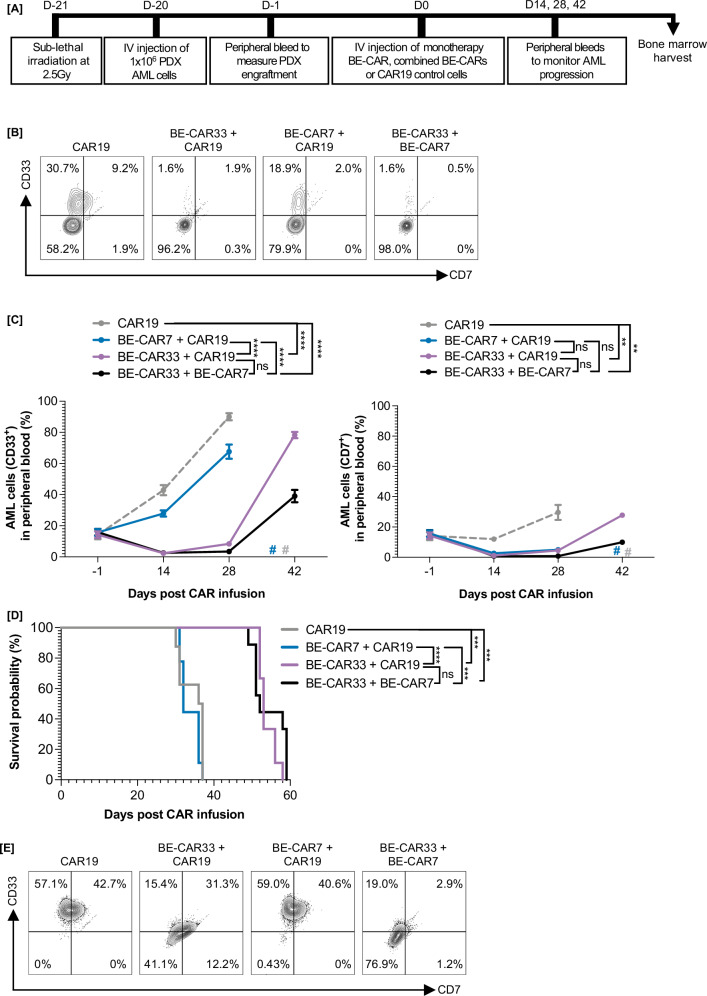
Removal of CD7 enables compatibility of BE-CAR7 and BE-CAR33 in a PDX model of AML. **A** 1×10^6^ CD33^high^CD7^low^ primary AML cells were injected into sub-lethally mice and monitored for disease engraftment through peripheral sampling for 3 weeks. Control CAR19 effectors, an antigen not expressed by AML or T cells, were injected alone or in combination with BE-CAR7 or BE-CAR33 to negate dose-dependent BE-CAR7 or BE-CAR33 effects when co-injected versus delivered in isolation. **B** Example flow cytometry and subsequent tracking **C** of peripheral CD33^+^ (left) and CD7^+^ (right) disease after treatment with 10 × 10^6^ CAR19^+^ T cells (*n* = 10) (from 10 × 10^6^ MNCs) or 10 × 10^6^ CAR19^+^ T cells and 6 × 10^6^ BE-CAR7^+^ T cells (*n* = 10), 10 × 10^6^ CAR19^+^ and 10 × 10^6^ BE-CAR33^+^ T cells (*n* = 10) or 10 × 10^6^ BE-CAR33 and 6 × 10^6^ BE-CAR7 T cells (*n* = 10) (from 20 × 10^6^ MNCs). # indicates animal death. ** *p* < 0.01, **** *p* < 0.0001 (one-way ANOVA of AUC with Tukey multiple comparison post-hoc between D-1 and D28). **D** Survival of mice treated with CAR19 T cells (*n* = 10), CAR19 and BE-CAR7 T cells (*n* = 10), CAR19 and BE-CAR33 T cells (*n* = 10), or BE-CAR33 and BE-CAR8 T cells (*n* = 10). *** *p* < 0.001, **** *p* < 0.0001 (Kaplan-Meir with Mantel-Cox test). **E** Example flow cytometry showing bone marrow CD33^+/-^CD7^+/-^ disease following BE-CAR treatment.

Strategies were also tested in vivo to examine the feasibility of using BE-CAR33 or BE-CLL1 T cells in combination with BE-CAR7 T cells, noting that the latter is already being applied in human studies against T-ALL, and can offer lymphodepleting effects as well as anti-leukaemia activity against CD7^+^ AML. For these experiments, BE-CAR33 or BE-CLL1 T cells were simultaneously base edited to remove CD7 to prevent targeting by BE-CAR7 T cells and both populations were also edited to remove *CD52* to permit co-infusion in the presence of alemtuzumab in future clinical applications. We first investigated the compatibility of co-infused CD7 edited BE-CAR combinations in our heterogenous cell line model of AML (Fig. S3J–M). In this study, BE-CAR33 and BE-CARCLL-1 effectors continued to clear antigen-expressing disease when infused alone or in combination with BE-CAR7 product. Next, we used a PDX murine model where CD33^high^ CD7^low^ primary AML cells were engrafted into 8-12 week-old NSG mice following sub-lethal (2.5 Gy) irradiation (Fig. 4A). Peripheral blood sampling confirmed AML engraftment three weeks later and effectors were injected in the following groups: 10 × 10^6^ CAR19^+^ control T cells alone (*n* = 9) or in combination with either 10×10^6^ BE-CAR33^+^ (*n* = 10) or 6 × 10^6^ BE-CAR7^+^ (*n* = 10), or 10 × 10^6^ BE-CAR33^+^ and 6 × 10^6^ BE-CAR7^+^ co-injected T cells (*n* = 10). Serial peripheral bleeds were performed on day 14, 28 and 42 post-effector infusion to track disease progression. By day 28, groups that received BE-CAR33 T cells alone or in combination with BE-CAR7 exhibited delayed disease progression compared to mice that received BE-CAR7 and/or CAR19 control cells (*p* < 0.0001) (Fig. 4B, C). Mice treated with BE-CAR33 alone or in combination with BE-CAR7 effectors demonstrated significantly prolonged survival compared with control groups (*p* < 0.001, *p* < 0.0001), (Fig. 4D), and bone marrow analysis confirmed clearance of the majority of CD33^+^ disease two weeks post co-infusion (Fig. 4E). Importantly, BE-CAR33 effectors continued to exert their anti-leukaemia effects when co-injected with BE-CAR7 cells, with no difference noted in the survival of either group, confirming successful evasion of fratricidal effects after removal of CD7. Residual bone marrow blasts were noted to be CD33^dim^CD7^low^ further demonstrating activity of the BE-CAR33 product (Fig. 4E). These findings together confirmed that anti-leukaemia effects of CD7 edited BE-CAR33 T cells were preserved and remained functional in the presence of BE-CAR7 T cells.

## Discussion

Children and adults with relapsed or refractory AML continue to face dismal long term survival outcomes with current therapy regimens [1]. CAR T cells offer a promising alternative but targeting of a single antigen may not be as effective as with B- or T-cell malignancies due to high levels of inter- and intra-patient phenotypic heterogeneity [15]. A paucity of leukaemia-specific antigens also remains problematic, and their expression inevitably overlaps with healthy haematopoietic or other tissues; for example, autologous and allogeneic CAR T-cell therapies targeting the interleukin-3 receptor (CD123) have been investigated in clinical trials, but with notable toxicities and a varied expression profile across age groups [20–22]. Other antigens of interest may also be present on precursors or mature granulocytes, macrophages and T cells, and myeloablative and lymphodepleting CAR T-cell effects can create protracted risks of cytopenia and opportunistic infections [23, 24]. In addition, disease heterogenicity suggests that CAR T-cell therapies against multiple leukaemia antigens may be needed to ensure complete coverage of disease profiles, including both childhood and adult AML. CAR T cells used in combination may address this whilst mediating disease clearance ahead of donor-derived stem-cell rescue.

CD33 is broadly expressed on AML blasts and leukaemic stem cells (LSCs) and has been targeted using the drug conjugated humanised anti-CD33 monoclonal antibody (mAb) Gemtuzumab ozogamicin (GO) [25, 26]. A number of alternative anti-CD33 antibody drug conjugates (ADCs), radiolabelled antibodies, bi-specific antibodies and CAR T cells carrying GO-derived binders have also been investigated clinically [27–30]. Qin et al. encountered toxicities in their murine studies and instead proposed a Lintuzumab-derived CAR T product with a CD28-CD3ζ signalling domain, and results from a corresponding clinical study in children and young adults suggested manageable toxicities with complete responses and myeloid aplasia reported in 2 out of 6 patients at higher doses [31, 32]. Our GO-derived BE-CAR includes 41BB-CD3ζ signalling domains and successfully inhibited AML in humanised mice without obvious toxicity, including when used in combination with other BE-CAR products. Considering extensive pre-existing clinical safety data from anti-CD33 antibody studies, BE-CAR33 has been adopted for early phase clinical investigation in an allogeneic off-the-shelf approach in children (NCT05942599). Clinical experience with similar autologous anti-CD33 CAR T-cell products have noted difficulties with apheresis harvests in heavily pre-treated patients [33]. Experience is limited to small series reports; Tambaro et al. treated 3 patients, describing cytokine release syndrome (CRS) and immune effector cell-associated neurotoxicity syndrome (ICANS), whilst Wang et al. reported transient blast reduction in a single patient [34]. Rapidly produced anti-CD33 CAR T cells with membrane bound IL-15 (mbIL-15) and a suicide switch have also been tested in a phase 1 clinical trial with documented objective responses in AML patients who received cells following lymphodepletion [35]. Recently, Applebaum et al. reported variant CD33 binders with rapamycin-regulated dimerization for controlled expression, and clinical endpoints were consistent with T-cell expansion alongside signs of anti-leukaemia activity [36].

CLL-1 is a type II transmembrane C-type lectin-like receptor that is often aberrantly expressed on AML blasts, as well as on LSCs [3, 37]. CLL-1 targeting CAR T cells have been documented in clinical case reports; Zhang et al. reported complete responses in a small number of children, including sustained morphologic remission following an allo-SCT [38–40]. Separately, six out of nine adults were reported to be in ongoing complete remission three months following treatment [41]. Our BE-CARCLL-1 product was designed using the anti-CLL-1 mAb clone M26 following promising reports from both studies. A separate CD28-CD3ζ CAR designed with the same anti-CLL-1 mAb clone is also under clinical investigation (NCT04219163) [42]. Targeting of CLL-1 alongside CD33 has been investigated, including through a self-cleaving P2A peptide sequence allowing for a 1:1 expression of both CAR constructs [43]. Six of nine patients were reported as MRD negative following treatment with this product and allo-SCT.

We also investigated the use of anti-CD7 CAR T cells as part of a combinatorial approach. CD7 is an Ig-superfamily molecule that is highly expressed on T cells NK cells and some AML cases, with anti-CD7 CAR T cells under clinical investigation to treat CD7^+^ disease [44]. We previously reported the generation of fratricide- and lymphodepletion-resistant universal BE-CAR7 T cells, developed originally to treat CD7^+^ T-cell malignancies [13]. Multiple studies, including one at our centre, have reported profound lymphodepletion and bone marrow aplasia post CAR T-cell infusion [45]. There are also reports of successful haploidentical-SCT without the need for additional myeloablative pre-conditioning post anti-CD7 CAR therapy [14]. We anticipate BE-CAR7 may offer dual benefits of targeting CD7^+^ disease and augmenting lymphodepletion and bone marrow conditioning ahead of bridging allo-SCT.

For BE-CAR33 and BE-CARCLL-1 products, the vector configuration included a sgRNA cassette embedded in the vector LTR as previously described for CAR19 products used against B-ALL [18]. Delivery of sgRNAs by electroporation along with BE mRNA also provided knock out effects for resistance to pre-conditioning serotherapy with anti-CD52 antibody (Alemtuzumab) and removed CD7, a shared T-cell antigen. We previously reported in-depth interrogations confirming on-target fidelity of these sgRNAs and mapped negligible off-target effects [13, 45].

Each BE-CAR product was able to efficiently inhibit respective antigen-expressing AML with concordant elevation of cytokines in vitro and anti-leukaemia potency in vivo in humanised murine models. Previous reports tested combined antigen targeting of MOLM-13, HL-60 and KG1a AML cell lines and noted improved survival following combined dosing with anti-CD33 and anti-CLL-1 CAR T cells compared to monotherapy options [42]. We used CRISPR/Cas9 mediated removal of CD33 and CLL-1 form HL-60 cells to generate a heterogenous model suitable for assessment of specific BE-CAR products. Monotherapy groups cleared target positive disease but succumbed to antigen negative disease, whilst combined infusions of BE-CAR33 and BE-CARCLL-1 cells were able to control disease progression. These effectors also evaded BE-CAR7 in vivo having been edited to remove CD7 expression. Furthermore, in a primary PDX AML model, CD33^high^CD7^low^ disease was effectively targeted by BE-CAR33 with longer overall survival even in the presence of BE-CAR7. Whilst this particular model could not assess direct BE-CAR7 activity against AML, the preservation of BE-CAR33 effects was important to establish following co-injection of both products. We envisage adoption of “universal” BE-CAR33, BE-CARCLL-1 and BE-CAR7 products for AML eradication or control ahead of allogeneic stem cell transplant. Deep preparative conditioning ahead of programmed transplant and donor-derived immunological reconstitution may offer valuable routes to treatment for hard-to-treat heterogenous disease.

Genome editing has unlocked opportunities for engineering of CAR T cells, and may be further exploited for modification of shared antigens on healthy HSCs to support persistence during anti-leukaemia therapy. CRISPR-mediated removal of CD33 on healthy HSCs is being investigated in combination with GO antibody therapy (NCT04849910), whilst base editing for disruption of FLT-3 and CD45 epitopes has been modelled to allow evasion of CAR-T effects [46–48]. Modification of autologous HSCs may be challenging in subjects who may harbour underlying cancer predispositions, or who have been heavily pre-treated. In the first instance, allogeneic universal donor BE-CAR T cells, alone or in combination, offer innovative routes to disease clearance and remission if planned carefully ahead of allo-SCT and donor derived reconstitution.

## Supplementary information

SUPPLEMENTAL MATERIAL

## Data Availability

The datasets generated during and/or analysed during the current study are available from the corresponding author on reasonable request.
