# Supplementary material for: Base edited “universal” donor CAR T-cell strategies for acute myeloid leukaemia
Source: Leukemia. 2025 Oct 1;39(12):2978–87. doi: 10.1038/s41375-025-02720-5 (PMC12634419; doi:10.1038/s41375-025-02720-5)
Supplement: Supplementary file 1 — SUPPLEMENTAL MATERIAL [file 41375_2025_2720_MOESM1_ESM.pdf]

Base edited "universal" donor CAR T cell strategies for acute myeloid leukaemia – supplemental methods and results

Authors

Renuka Kadirkamanathan[1]*, renuka.kadirkamanathan.14@ucl.ac.uk

Christos Georgiadis[1]*, christos.georgiadis@ucl.ac.uk [corresponding author]

Arnold Kloos[2], kloos.arnold@mh-hannover.de

Akshay Joshi[1], akshay.joshi@ucl.ac.uk

Annie Etuk[1], annie.etuk@gosh.nhs.uk

Roland Preece[1], roland.preece.13@ucl.ac.uk

Oliver Gough[1], o.gough@ucl.ac.uk

Axel Schambach[3, 4], schambach.Axel@mh-hannover.de

Martin Sauer[5], sauer.Martin@mh-hannover.de

Michael Heuser[2,6], heuser.michael@mh-hannover.de

Waseem Qasim[1], w.qasim@ucl.ac.uk

1 UCL Great Ormond Street Institute of Child Health, London, UK

2 Department of Hematology, Hemostasis, Oncology, and Stem Cell Transplantation, Hannover Medical School, Hannover, Germany

3 Institute of Experimental Hematology, Hannover Medical School, Hannover, Germany

4 Division of Hematology/Oncology, Boston Children's Hospital, Harvard Medical School, Boston, MA, USA

5 Department of Pediatric Hematology and Oncology and Blood Stem Cell Transplantation, Hannover Medical School, Hannover, Germany

6 Department of Internal Medicine IV, University Hospital Halle (Saale), Martin-Luther-University Halle-Wittenberg, Halle, Germany
* The authors contributed equally.

Contents **Page**

Methods	2
Figure S1 Manufacture of BE-CAR T cells	6
Figure S2 Characterisation of BE-CAR T cells	8
Figure S3 Assays and models for functional assessments of BE-CAR T cells	10
Figure S4 BE-CAR clearance of heterogenous AML	13
References	18

Supplemental methods

Flow cytometry

AML cell lines and samples from *in vivo* studies were blocked with Human TruStain FcX (BioLegend) when required for staining. Samples were acquired using a CytoFLEX cytometer (Beckman Coulter, High Wycombe, UK), CyAn™ ADP analyser (Beckman Coulter), BD® FACSymphony™ cytometer (BD, Franklin Lakes, NJ, USA) or the BD® LSR II flow cytometer (BD). Data was analysed using FlowJo™ software v10 (TreeStar Inc, Ashland, OR, USA). The following antibodies were used: CD45 (HI30 clone, Miltenyi (Bergisch Gladbach, Germany), HI30/5B1 clone, BioLegend (San Diego, CA, USA)), CD8 (REA734 clone, Miltenyi), CD4 (M-T466 clone, Miltenyi, SK3 clone, BioLegend), CD2 (LT2 clone, Miltenyi, RPA-2.10 clone, BioLegend), $TCR_{\alpha/\beta}$ (BW242/412 clone and REA-652 clone, Miltenyi, IP26 clone, BioLegend), CD52 (HI186 clone, BioLegend), CD7 (CD7-6B7 clone, BioLegend), CD45RA (REA1047 clone, Miltenyi), CD62L (145/15 clone, Miltenyi), CCR7 (FR 11-11E8 clone, Miltenyi), PD-1 (EH12.2H7 clone, BioLegend), TIM-3 (7D3 clone, BioLegend), LAG-3 (T47-530 clone, BioLegend), BTLA (MIH26 clone, BioLegend, J168-540 clone, BD), CD69 (FN50 clone, BioLegend), CLL-1 (50C1

clone, BioLegend), CD33 (WM53 clone, BioLegend). To assess cell surface CAR expression, samples were stained with biotin-conjugated goat anti-mouse IgG F(ab)$_2$ fragment (Jackson Immunoresearch, Stratech Scientific Limited, Ely, United Kingdom) followed with PE-conjugated streptavidin (Miltenyi). The following isotype controls were used: anti-mouse IgG1κ (MOPC-21 clone) and anti-mouse IgG2aκ (MOPC-173 clone), both from BioLegend.

SpCas9- and BE3-mediated genome editing

The following sgRNAs, comprising a 20-nt protospacer sequence, 80-nt CRISPR scaffold and 2'O-methyl 3' phosphorothioate modifications, were supplied by Synthego (California, US) by automated solid-phase synthesis and eluted into nuclease-free Tris-EDTA buffer. sgRNAs were employed against *TRBC* (exon 1 - tryptophan to pmSTOP) 5' CCCACCAGCTCAGCTCCACG 3', *CD7* (exon 2 - glutamine to pmSTOP) 5' CACCTGCCAGGCCATCACGG 3' and *CD52* (exon 1 - splice site disruption) 5' GGTTATGGTACAGGTAAGAG 3', *CD33* (exon 1 - pmSTOP) 5' TGACAACCAGGAGAAGATCG 3' and *CLEC12A* (exon 1 – double stranded break formation) 5' TGCTGGACGCCATACATGAG 3'. mRNA for codon optimised SpCas9 (coSpCas9) or codon optimised BE3 (coBE3) was supplied by TriLink BioTechnologies (San Diego, CA, USA) and BioNTech (Mainz, Germany) respectively.

Molecular quantification of on-target genome editing

Genomic DNA was extracted using a DNeasy Blood and Tissue Kit (QIAGEN, Hilden, Germany), and 400-800bp fragments including the protospacer sequence were amplified via PCR using the following primers: *TRBC* fwd 5' AGGTCGCTGTGTTTGAGC 3' and *TRBC* rev 5' CTATCCTGGGTCCACTCGTC 3', *CD7* fwd 5' TAGGTGAGACCGCCCCTCC 3' and *CD7* rev 5' GGGACCCTGAGAAGTCGATGC 3', *CD52* fwd 5'

CTACCAAGACAGCCACGAAGAT 3’ and *CD52* rev 5’ TGCTCTCAGGAGAGAAGGCTG 3’. PCR products were purified using a QIAQuick PCR purification kit (QIAGEN) and sent for Sanger sequencing (Eurofins Genomics, Luxembourg City, Luxembourg). Rates of on-target C>T conversion were calculated using EDITR software (https://moriaritylab.shinyapps.io/editr_v10/).

Quantification of vector copy number

Vector copy number was quantified by ddPCR For HIV psi and human albumin using FAM and HEX respectively and a QX200 AutoDG Droplet Digital PCR system (BioRad) as described previously [1].

Cytometric bead array for cytokine release assays

Target cell lines were co-cultured with BE-CAR T cells or untransduced T cells at an E:T of 1:1, each at $1x10^6$ per mL, for 16 hours at 37°C in 20% IMDM. Supernatants were harvested and sample preparation and acquisition was performed using the Human Th1/2/17 CBA kit (BD) and a BD® LSR II flow cytometer, respectively. Data was then analysed using BD FCAP Array software v3.

Generation of GFP expressing cell lines

Cells were with pCCL lentiviral vector encoding a GFP-P2A-luciferase sequence and enriched through fluorescence activated cell sorting (FACS) using a FACSAria™ cell sorter (BD).

Generation of heterogenous target cell lines

HL-60 cells were edited using sgRNA at 10μg/mL and either SpCas9 at 100μg/mL or BE3 at 50μg/mL, which were delivered via electroporation using a Lonza 4D-Nucleofector™ (EN138 pulse code). Cells were recovered at 30°C for 16 hours and returned to 37°C. Knockout of CD33 and CLL-1 was confirmed through flow cytometry and cells were enriched by FACS using a FACSAria™ cell sorter (BD).

Guidelines for use of PDX model

The PDX mouse model, established at Hannover Medical School, was conducted with approval from the Lower Saxony state office for consumer protection, Oldenburg, Germany. Mice were kept under pathogen-free conditions at the central animal laboratory of Hannover Medical School.

Supplemental results

Figure S1 Manufacture of BE-CAR T cells

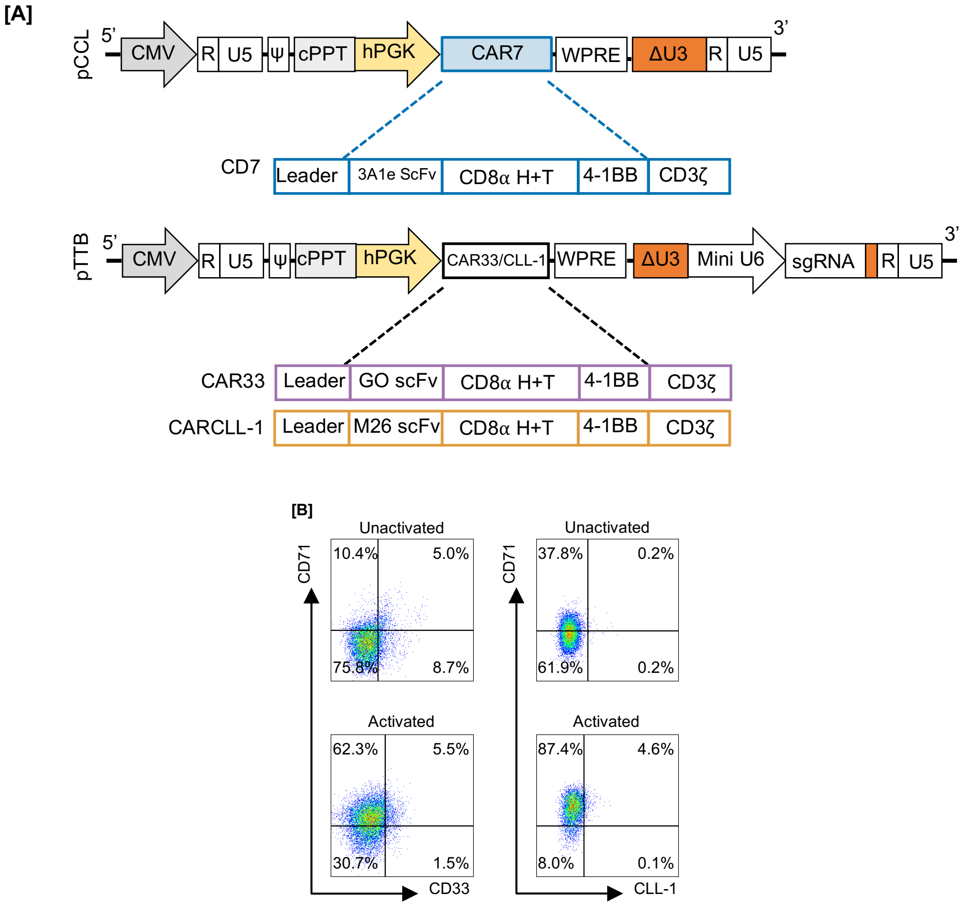

[A] pCCL and pTTB lentiviral vector transfer plasmids for the manufacture of BE-CAR7 (top) and BE-CAR33 or BE-CARCLL-1 (bottom) T cells. The CAR-encoding gene was under the control of an internal human phosphoglycerate kinase (hPGK) RNA polymerase II promoter. For pTTB-CAR33 and pTTB-CARCLL-1 plasmids, a sgRNA expression cassette against *TRBC* (with a modified c+5 scaffold) was present in the 3' LTR under the control of an internal "mini U6" RNA polymerase III promoter described previously [2]. CMV; cytomegalovirus. **[B]** Representative flow cytometry plot showing minimal cell surface expression of CD33 (left) and CLL-1 (right) on unactivated T cells (top) and T cells 4 days after activation with TransAct (bottom). CD71 was used as a marker for T cell activation. CD33 and CLL-1 gates were established using appropriate isotype controls.

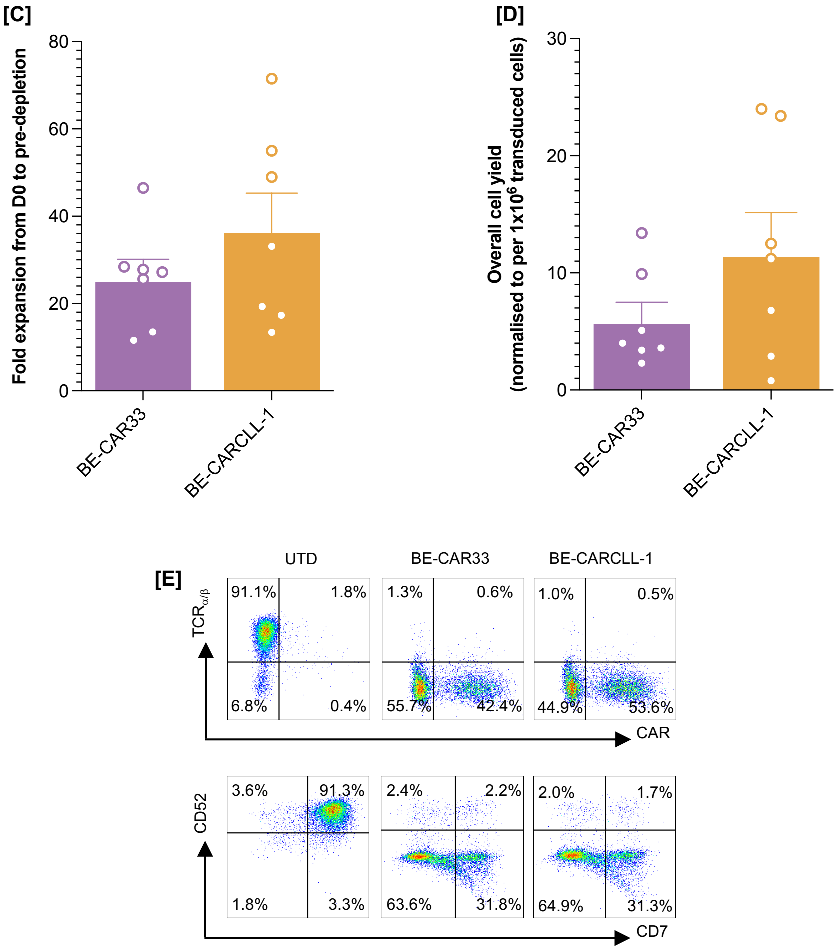

[C] High levels of fold expansion and overall cell yield **[D]** were obtained during BE-CAR33 and BE-CARCLL-1 T cell manufacture**.** **[E]** Representative flow cytometry plot of $CD45^{+}$ untransduced and end-of-manufacture BE-CAR33 and BE-CARCLL-1 T cells displaying surface CAR and/or TCRαβ (top row) and CD7 and/or CD52 (bottom row) expression following transduction followed by electroporation with sgRNA sequences against *CD7*, *CD52* and *TRBC*.

Figure S2 Characterisation of BE-CAR T cells

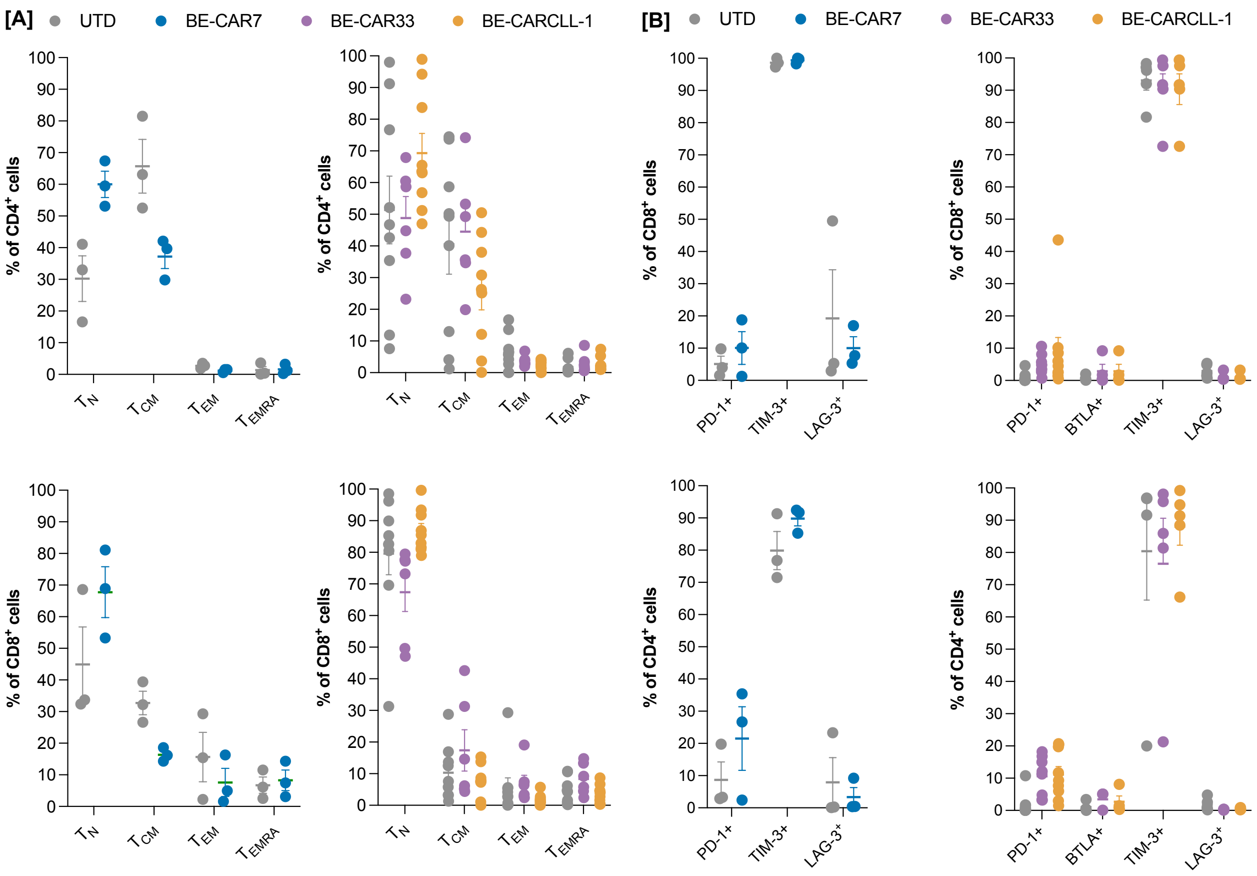

[A] $CD4^+$ and $CD8^+$ untransduced T cells (n=3, n=9), BE-CAR7 (n=3) (left), and BE-CAR33 (n=6) as well as BE-CARCLL-1 (n=9) T cells (right) displaying combinations of CD62L and/or CD45RA expression profiles associated with T_N, T_{CM}, T_{EM} or T_{EMRA} subsets. **[B]** $CD4^+$ (top) and $CD8^+$ (bottom) untransduced cells, BE-CAR7 (left), as well as BE-CAR33 and BE-CARCLL-1 cells (right) displaying cell surface PD-1 (n=3, n=8, n=9 across respective BE-CAR products), BTLA (n=4 across BE-CAR33 and BE-CARCLL-1 only), TIM-3 (n=3, n=5, n=5 across respective BE-CAR products), and LAG-3 (n=3, n=5, n=5 across respective BE-CAR products).

[C]

Product	Donor 1	Donor 2	Donor 3	Donor 4	Donor 5	Donor 6
**BE-CAR33**	1.59	1.58	6.04	3.85	1.06	6.89
**BE-CARCLL-1**	3.57	4.19	4.00	3.72	0.71	2.84

[D]

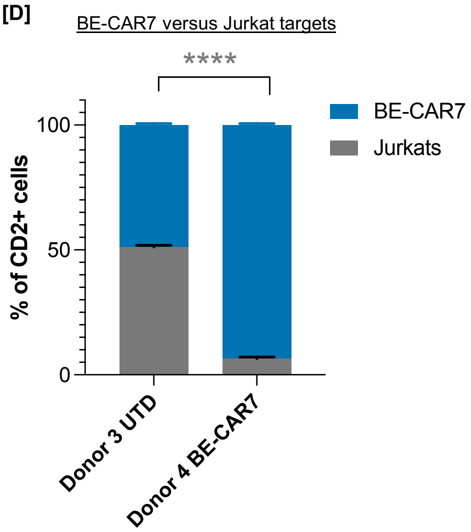

[C] VCN analysis of BE-CAR33 and BE-CARCLL-1 products measured 3.5±1.0 and 3.2±0.5 average copies per cell, respectively. **[D]** Cytotoxicity of BE-CAR7 effectors used in Fig. 1H was confirmed through the clearance of $CD7^{+}GFP^{+}$ Jurkat targets after co-culture for 16 hours at an E:T of 1:1. Data is shown for one BE-CAR7 donor measured in triplicate. **** $p < 0.0001$ (unpaired T test).

Figure S3 Assays and models for functional assessments of BE-CAR T cells

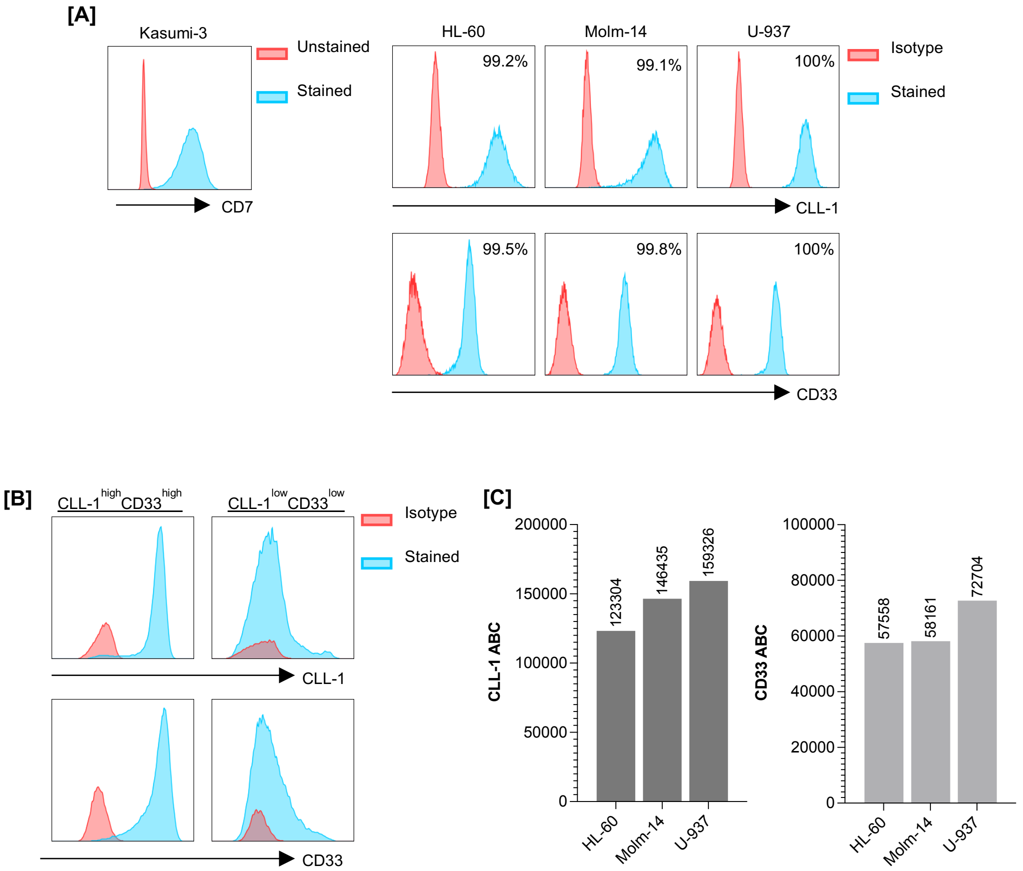

[A] Kasumi-3 AML cells demonstrating cell surface CD7, detected using the CD7-6B7 antibody clone, and HL-60, Molm-14 and U-937 AML cell lines demonstrating cell surface CLL-1 and CD33 expression detected using 50C1 or P67.6 antibody clones respectively. Analysis of HL-60, Molm-14 and U-937 cells was normalised using an isotype control. **[B]** Primary AML samples displaying high or low levels of CLL-1 (detected using the 50C1 antibody clone) and CD33 (detected using the P67.6 antibody clone), referred to as CLL-1^{high}CD33^{high} and CLL-1^{low}CD33^{low} respectively. **[C]** Antibody binding capacity (ABC) of the 50C1 anti-CLL-1 antibody clone (left) and P67.6 anti-CD33 antibody clone (right) on HL-60, Molm-14 and U-937 AML cell lines.

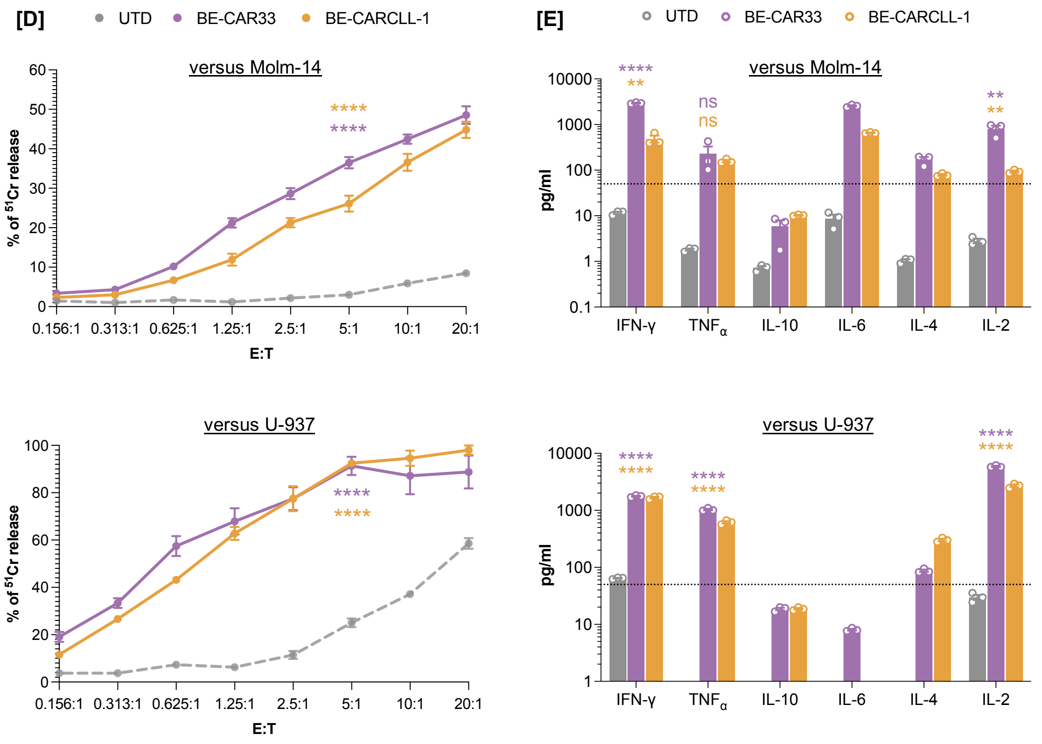

[D] Normalised ^{51}Cr release from pre-labelled Molm-14 (top) or U-937 (bottom) targets when co-cultured with BE-CAR33 or BE-CARCLL-1 for 4 hours at E:Ts ranging from 20:1 to 0.156:1. Untransduced cells were included to demonstrate target lysis in the absence of a CAR. **** $p < 0.0001$ (one-way ANOVA with Tukey multiple comparison post-hoc). **[E]** Cytokines released from untransduced BE-CARCLL-1 or BE-CAR33 T cells when co-cultured with Molm-14 (top) or U-937 (bottom) at an E:T of 1:1 for 16 hours. Cytokine release below 50 pg/mL was considered background (dotted line). Data is shown for one BE-CAR33/BE-CARCLL-1 donor measured in triplicate. ** $p < 0.01$, **** $p < 0.0001$ (one-way ANOVA with Tukey multiple comparison post-hoc).

[F]

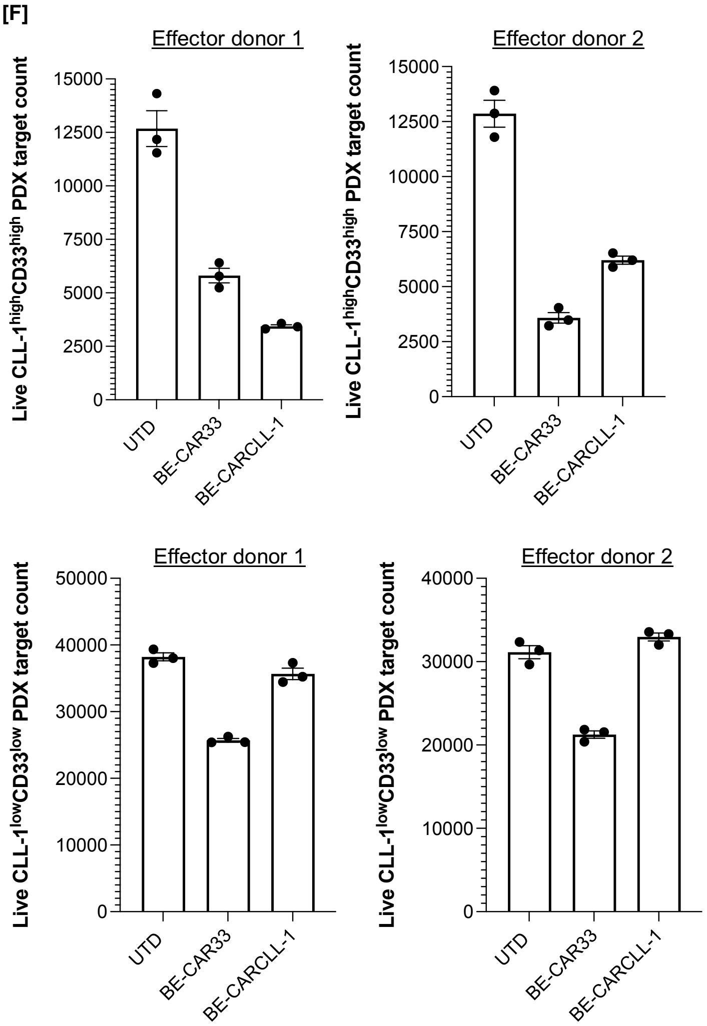

[F] BE-CAR33 and BE-CARCLL-1 T cells effectively cleared CD33^{high}CLL-1^{high} primary AML targets after 16-hour co-culture at an E:T of 10:1. Effects were less robust against CD33^{low}CLL-1^{low} primary AML for BE-CARCLL-1. Data is shown for two BE-CAR donors, each measured in triplicate.

Figure S4 BE-CAR clearance of heterogenous AML

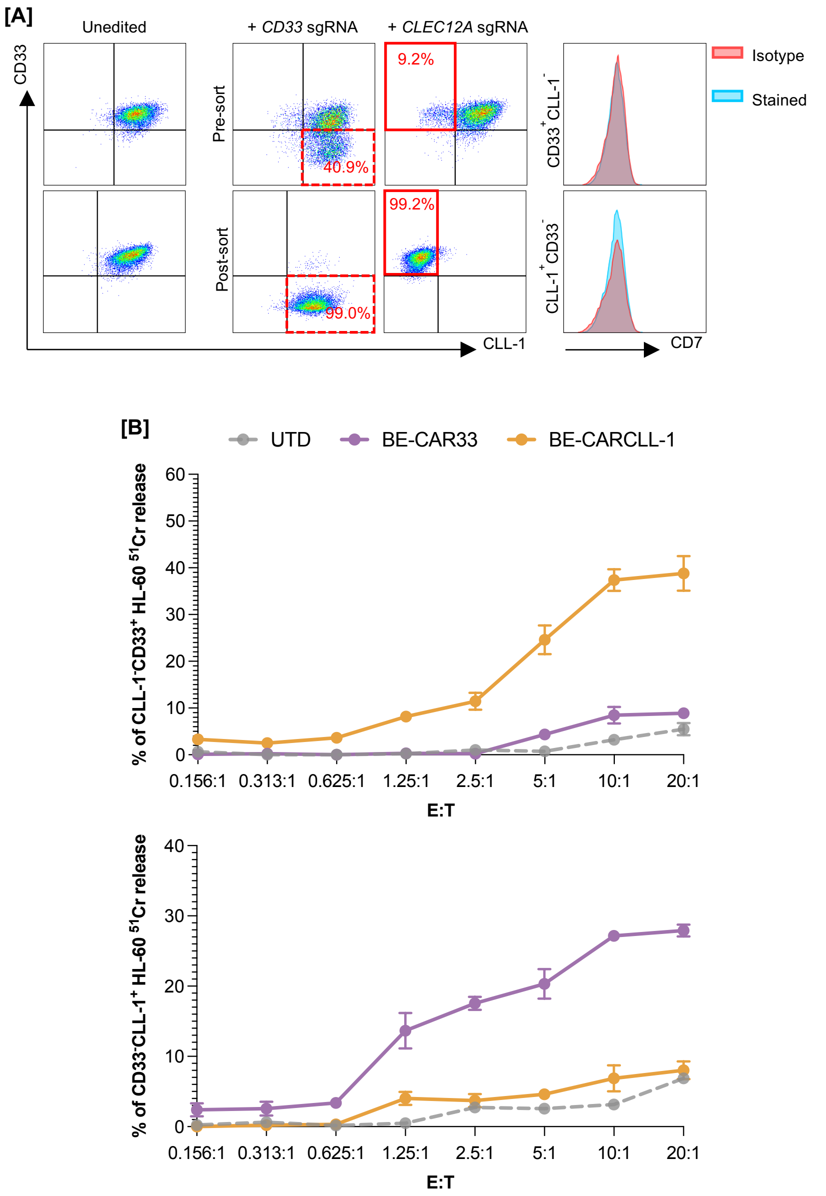

[A] (Left) HL-60 cells displaying cell surface expression of CD33 and/or CLL-1 4 days post electroporation with mRNA encoding coSpCas9 or coBE3, and sgRNA sequences against *CLEC12A* (CLL-1) or *CD33*, respectively, and FACS-assisted enrichment for respective phenotypes. Cells with CLL-1$^-$CD33$^+$ and CD33$^-$CLL-1$^+$ phenotypes are highlighted in solid and dashed boxes respectively. (Right) CD7 is absent from HL-60 cells. **[B]** Normalised ^{51}Cr release from pre-labelled CD33$^+$CLL-1$^-$ (top) and CLL-1$^+$CD33$^-$ (bottom) HL-60 targets when co-cultured with

untransduced cells, BE-CAR33 or BE-CARCLL-1 T cells for 4 hours at E:Ts ranging from 20:1 to 0.156:1.

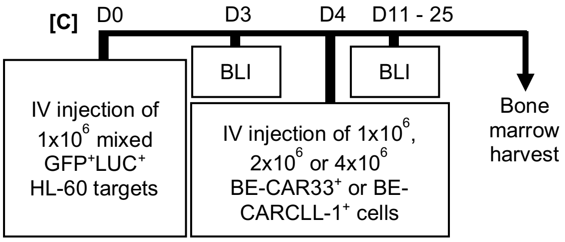

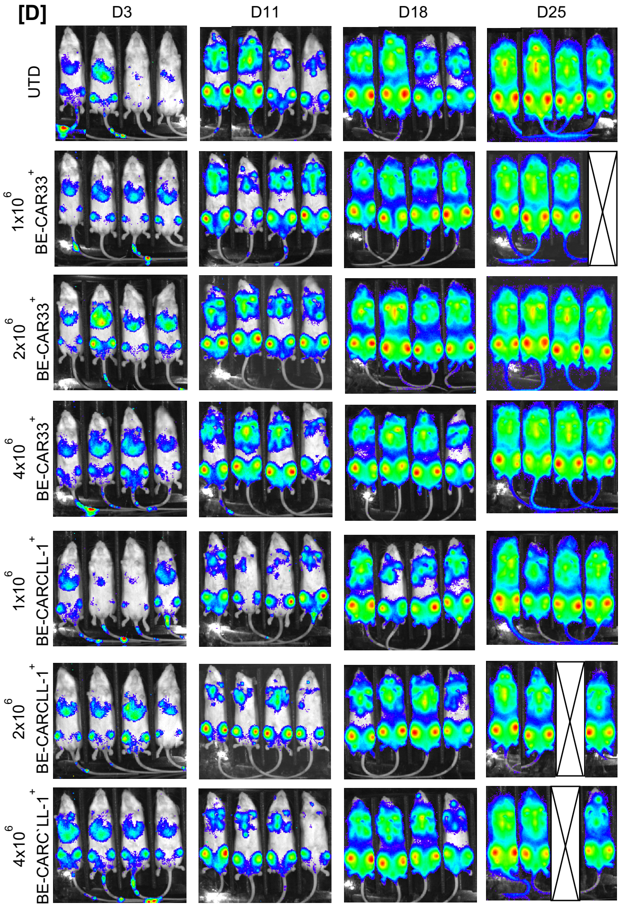

[C] Humanised xenograft model of heterogenous AML with GFP$^+$LUC$^+$ variants of CRISPR-engineered CD33$^+$CLL-1$^-$ and CD33$^-$CLL-1$^+$ HL-60 cell lines was used to assess dose-dependent effects of BE-CAR monotherapies. **[D]** Bioluminescent signals of mice that received 1x10^6 CD33$^{-/+}$CLL-1$^{-/+}$ HL-60 targets on day 0, and differing BE-CAR33$^+$ or BE-CARCLL-1$^+$ monotherapy doses (from a maximum of 10x10^6 MNCs) on day 4. Data for mice that received combined BE-CARs is shown in Fig. 3B.

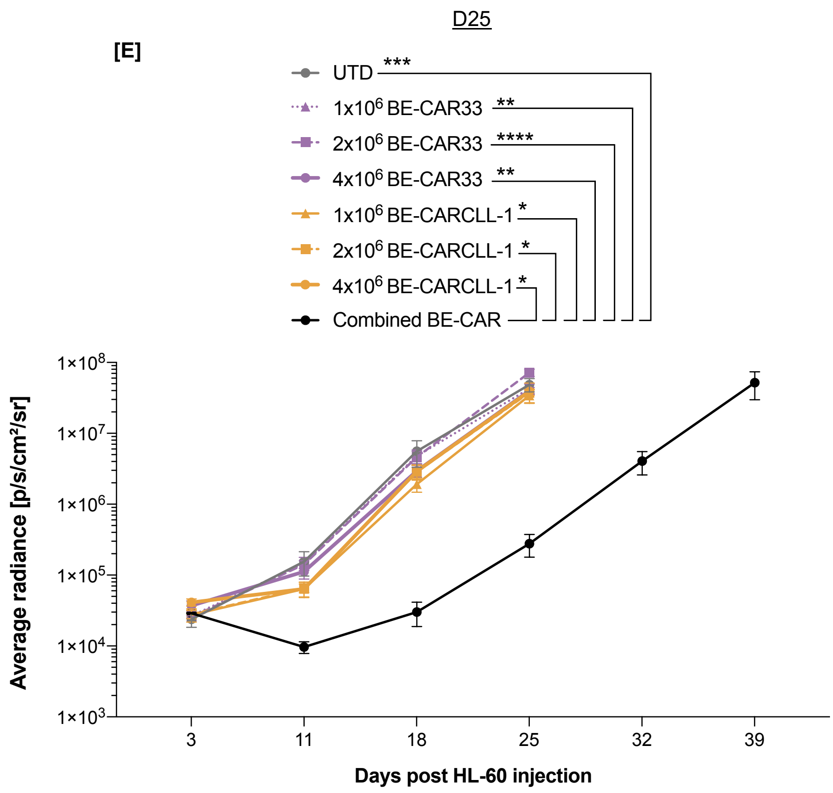

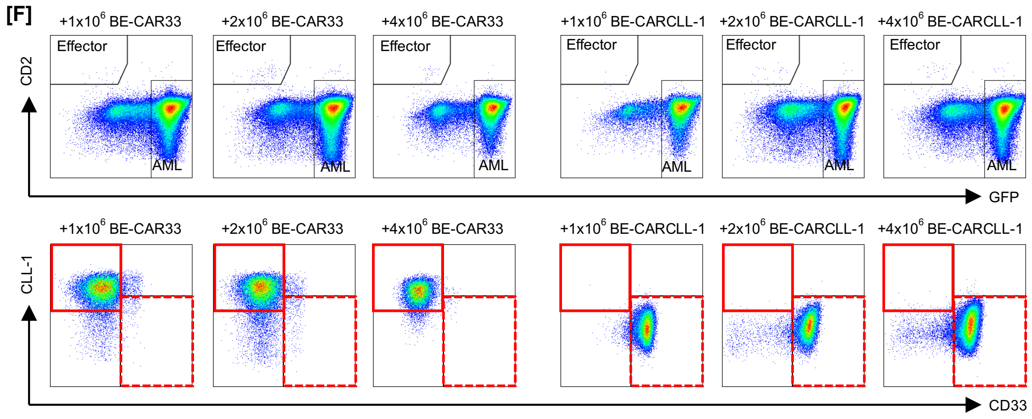

[E] Average radiance values (p/s/cm^2/sr) of mice injected with 1x10^6, 2x10^6 or 4x10^6 CAR$^+$ monotherapy effectors (n=4). BLI for mice treated with combined BE-CAR effectors is included in Fig. 3B). * p<0.05, ** p<0.01, *** p<0.001, **** p < 0.0001 (one-way ANOVA with Tukey multiple comparison post-hoc). **[F]** (Top) CD45$^+$CD2$^+$ effectors and CD45$^+$GFP$^+$ AML cells detected in bone marrow of mice from each monotherapy dose group. (Bottom) CD45$^+$GFP$^+$ AML cells detected in the bone marrow of mice from each monotherapy dose group displaying a CD33$^+$CLL-1$^-$, CD33$^-$CLL-1$^+$ or CD33$^-$CLL-1$^-$ phenotype. Cells with CLL-1$^-$CD33$^+$

and CD33^{-}CLL-1^{+} phenotypes are highlighted in solid and dashed boxes respectively.

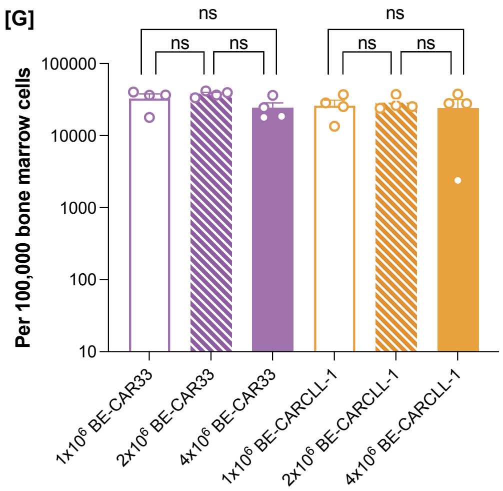

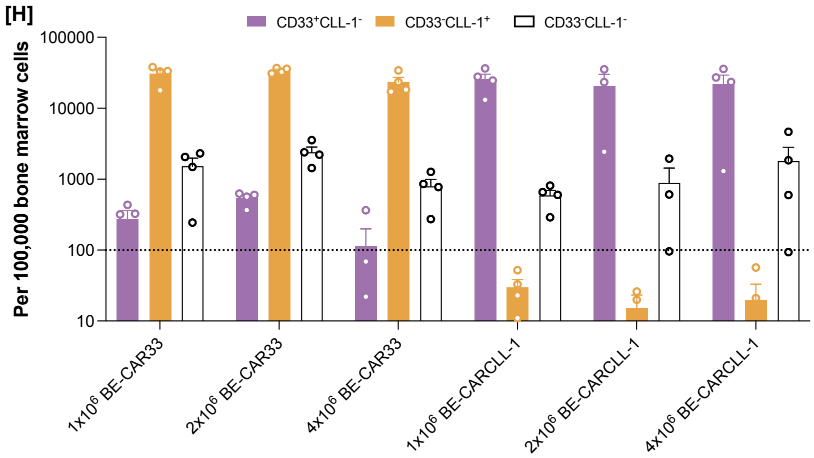

[G] Similar CD45^{+}GFP^{+} target cell counts were detected in bone marrow of mice from each monotherapy dose group suggested no dose related effects across this range. p=ns, (one-way ANOVA with Tukey multiple comparison post-hoc). **[H]** Normalised numbers of CD45^{+}GFP^{+} AML cells displaying a CD33^{+}CLL-1^{-} or CD33^{-}CLL-1^{+} phenotype detected in bone marrow of mice from each BE-CAR33 and BE-CARCLL-1 monotherapy dose group, respectively, consistent with antigen specific as opposed to dose-dependent BE-CAR effects. Limit of quantification is shown at 100 events per 100,000 bone marrow cells.

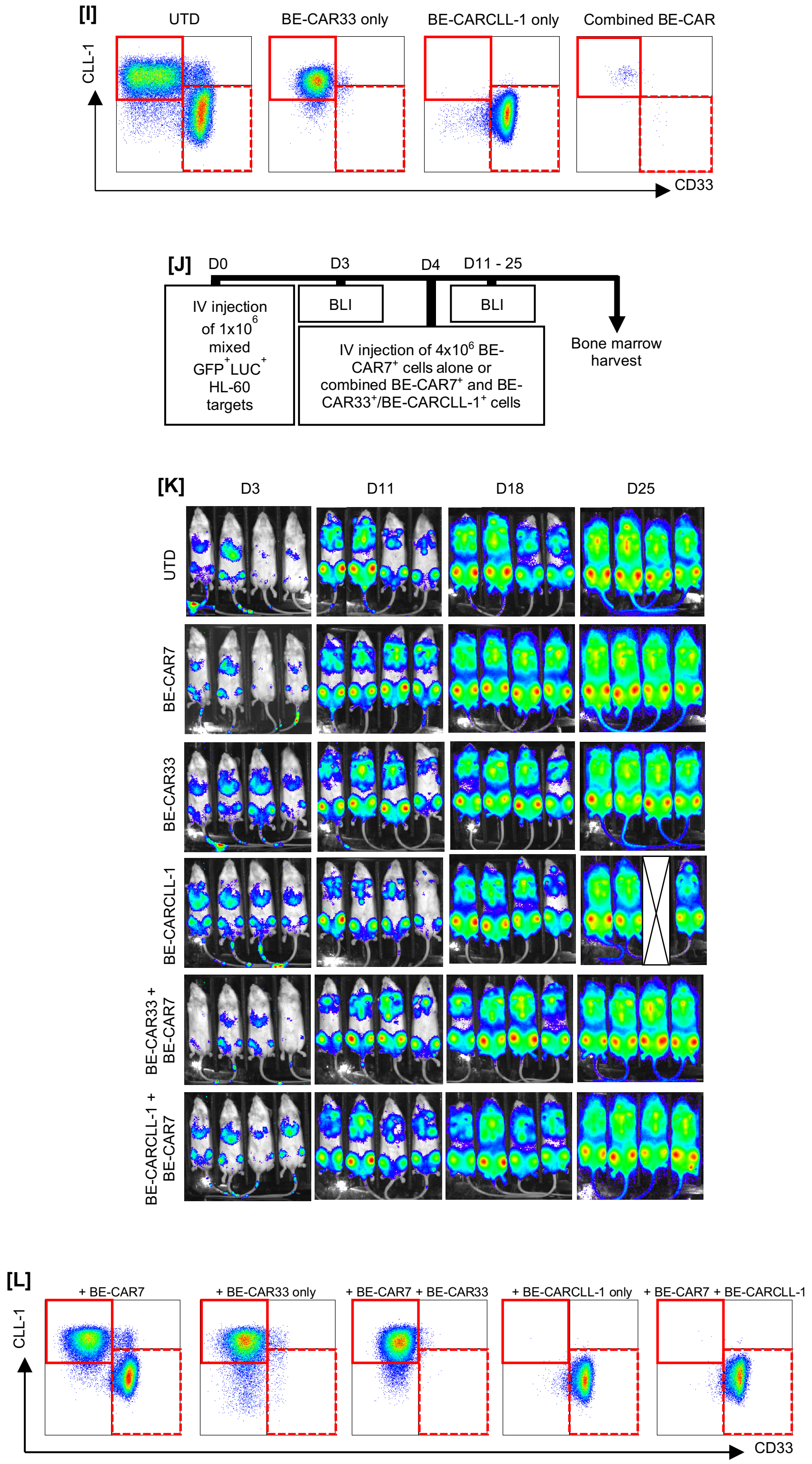

[I] CD45^{+}GFP^{+} AML cells detected in bone marrow of mice treated with UTD cells, BE-CARCLL- 1 or BE-CAR33 T cells in isolation, or in combination, displaying cell

surface CD33 or CLL-1 expression. **[J]** Humanised xenograft model of heterogenous AML with GFP$^+$ variants of CRISPR-engineered CD33$^+$CLL-1$^-$ and CD33$^-$CLL-1$^+$ HL-60 cell lines used to determine co-infusion compatibility between BE-CAR7 and CD7 edited BE-CAR33 or BE-CARCLL-1 effectors. **[K]** Bioluminescent signals from mice that received 1x10^6 CD33$^{-/+}$CLL-1$^{-/+}$HL-60 targets on day 0, and 4x10^6 BE-CAR7$^+$ cells alone or co-injected (2x10^6) with 2x10^6 BE-CAR33$^+$ or BE-CARCLL-1$^+$ cells (from a maximum of 10x10^6 MNCs). **[L]** CD45$^+$GFP$^+$ AML cells detected in the bone marrow of mice treated with BE-CAR7 alone and co-injected groups with CD33$^+$CLL-1$^-$ or CD33$^-$CLL-1$^+$ phenotypes.

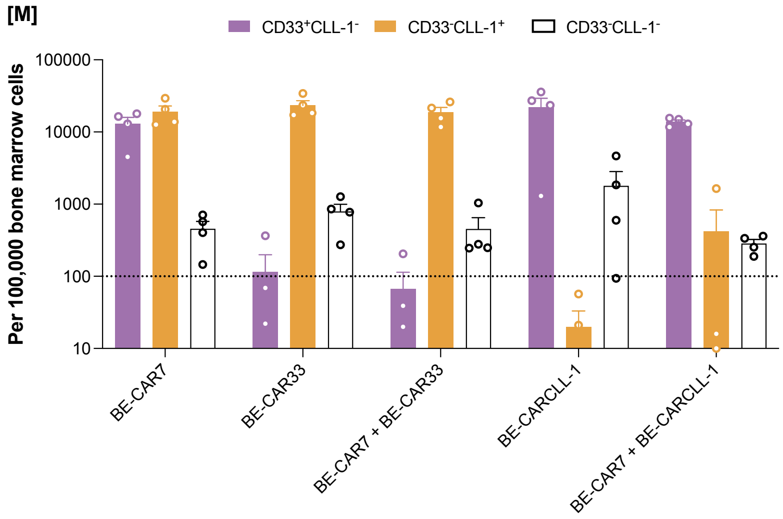

[M] CD7-edited BE-CAR33 and BE-CARCLL-1 effectors continued to clear CD33$^+$CLL-1$^-$ and CLL-1$^+$CD33$^-$ AML cells, respectively, when co-injected with BE-CAR7 cells. Limit of quantification is shown at 100 events per 100,000 bone marrow cells

1 . Chiesa R, Georgiadis C, Syed F, Zhan H, Etuk A, Gkazi SA *et al.* Base-Edited CAR7 T Cells for Relapsed T-Cell Acute Lymphoblastic Leukemia. N Engl J Med 2023; 389: 899–910.

2 . Preece R, Georgiadis C, Gkazi SA, Etuk A, Christi A, Qasim W. ‘Mini’ U6 Pol III promoter exhibits nucleosome redundancy and supports multiplexed coupling of CRISPR/Cas9 effects. Gene Ther 2020; 27: 451–458.
